# Sex variation in colorectal cancer mortality: trends and implications for screening

**DOI:** 10.1093/eurpub/ckad029

**Published:** 2023-02-27

**Authors:** Gavin R C Clark, Callum G Fraser, Judith A Strachan, Robert J C Steele

**Affiliations:** Public Health Scotland, Edinburgh, UK; Centre for Research into Cancer Prevention and Screening, University of Dundee, Dundee, UK; Blood Sciences and Scottish Bowel Screening Laboratory, Ninewells Hospital and Medical School, Dundee, UK; Centre for Research into Cancer Prevention and Screening, University of Dundee, Dundee, UK

## Abstract

**Background:**

Colorectal cancer (CRC) screening using faecal tests reduces disease-specific mortality. To investigate mortality and its association with sex, rates in women and men, and in different age ranges, were examined, before and after screening began in Scotland.

**Methods:**

From 1990–99, no structured screening existed. Three pilots ran from 2000 to 2007 and subsequent full roll-out completed in 2009. Crude mortality rates for 1990–2020 were calculated relative to Scottish population estimates, and age–sex standardized rates calculated for all, pre-screening (<50 years), screening (5–74 years) and post-screening (>74 years) age ranges.

**Results:**

CRC mortality declined from 1990 to 2020, but not linearly, and differed between sexes. In women, 1990–99 showed a steady decline [average annual percentage change (AAPC): −2.1%, 95% confidence interval (CI): −2.8% to −1.4%], but a less marked decline after 2000 (AAPC: −0.7%, 95% CI: −0.9% to −0.4%). In men, no clear decline was seen from 1990 to 1999 (AAPC: −0.4%, 95% CI: −1.1% to 0.4%), but mortality declined from 2000 to 2020 (AAPC: −1.7%, 95% CI: −1.9% to −1.5%). This pattern was exaggerated in the screening age ranges. For 2000–20, the overall reduction in mortality was less in women and in the screening age range. In the post-screening age range, reductions were smaller, but an increase was seen in the pre-screening age range, greater in women.

**Conclusions:**

CRC mortality fell during 1990–2020, but the decline differed markedly between sexes, indicating a larger beneficial effect of screening on CRC mortality in men compared to women: use of different thresholds for the sexes might lead to equality.

## Introduction

Randomized controlled trials (RCT) of colorectal cancer (CRC) screening using guaiac faecal occult blood tests (gFOBT) have consistently demonstrated significant reductions in disease-specific mortality,^1^ and this evidence has led to the introduction of screening programmes using tests for the presence of faecal haemoglobin (f-Hb) in many countries. Almost ubiquitously, gFOBT has been replaced by faecal immunochemical tests (FIT) owing to their improved performance characteristics,^2^ and there is every reason to expect the decrease in mortality to be maintained or even surpassed.

However, despite the fact that both women and men are invited in CRC screening programmes, there is now substantial evidence that women may benefit less than men from such programmes when they are based on the detection of f-Hb using a single f-Hb concentration threshold. Uptake of such screening is higher in women; however, this does not necessarily translate to improved outcomes for women, as their CRC detection rate is lower than that for men.^3^^,^^4^ To take a specific example, in the Scottish Bowel Screening Programme (SBoSP), after the replacement of gFOBT with FIT at a threshold of ≥80 µg Hb/g faeces, 0.09% of women (*n* = 307 919) and 0.15% of men (*n* = 279 530) screened were found to have CRC.^3^ This may be partially explained by the higher incidence in men; however, women are also more likely than men to be diagnosed with interval CRC (i.e. CRC detected within 2 years of a ‘negative’ screening test result),^5^^,^^6^ and more women than men are found to have CRC after an acute presentation to Accident and Emergency.^7^ Further, we have published data on the impact of screening participation on CRC incidence, with Supplementary material showing a more modest reduction in cumulative incidence for women participating in the screening programme than men.^8^

We have recently found that screen-detected CRC in women is associated with lower f-Hb concentrations than men.^9^ This indicates that screening using f-Hb has lower sensitivity for CRC in women than in men and it might be expected that the effect of screening on CRC mortality would be less in women than in men; indeed, the results of long-term follow-up of the Minnesota trial of gFOBT screening support this conclusion.^10^ Further confirmation comes from a recent study, showing that, in a retrospective cohort study on the FIT-based screening programme in the Italian region of Emilia-Romagna during the period 2005–16, following 707 751 individuals, CRC mortality was reduced by 54% and 75%, respectively, in women and men who participated regularly.^11^

For a CRC screening programme to fulfil its public health aim, there should be a demonstrable effect on disease-specific mortality at a whole population level, but there is a remarkable paucity of relevant data in the literature. Recently, a consortium of European cancer and death registries has published data on CRC incidence and mortality and shown consistent mortality reductions in countries with long standing screening programmes.^12^ However, there was no discussion of sex differences, and although the data showed reductions in mortality in women, this was restricted to countries that employed opportunistic colonoscopy screening.^13^

Therefore, to investigate the effect of a national f-Hb-based CRC screening programme on population CRC-specific mortality and to assess if any effects are associated with sex and differ between men and women, we examined the CRC mortality rates in both women and men and in different age ranges, stratified by sex and age-group, during the decade before, and then since, the introduction of f-Hb screening in Scotland.

## Methods

From 1990 to 1999, there was no structured CRC screening in Scotland, but comprehensive national data on mortality were collected by National Records Scotland (NRS). The SBoSP was initiated by a demonstration pilot running from 2000 to 2002 using biennial gFOBT (‘hema-screen’, Immunostics, Inc., Ocean, New Jersey, USA) in the 50–69 year age range in three territorial NHS Boards, which encompassed ∼25% of the Scottish population, following the screening strategy used in the Nottingham RCT.^1^ After a further two pilot screening rounds, roll-out across the whole of Scotland, still using initial gFOBT, but for a 50–74 year age range, started in July 2007 and was complete by December 2009. The two-tier reflex algorithm used has been described in detail.^14^ In November 2017, the SBoSP adopted a single quantitative FIT (HM-JACKarc, Minaris Medical Co., Ltd, Tokyo, Japan) as the screening test at a threshold of ≥80 µg Hb/g faeces.^3^ No exclusions are made from the invitations to participate in the SBoSP, save for those specifically requesting not to be invited.

Data on number of deaths where the underlying cause was attributed as CRC are gathered by NRS and published annually on the Public Health Scotland (PHS) website (Adapted from Cancer Mortality, PHS, 2021, licenced under the Open Government Licence: https://www.nationalarchives.gov.uk/doc/open-government-licence/version/3/).^15^ Data were available in 5-year age-bands and by sex.

Crude mortality rates were calculated relative to the NRS estimates of the Scottish population,^16^ and age–sex standardized rates and age-standardized rates for each sex derived using the 2013 European Standard Population. Age–sex standardized rates were calculated using the full age range, and then separately for the pre-screening (<50 years), screening (50–74 years) and post-screening (>74 years) age ranges. 95% confidence intervals (95% CIs) were calculated for these rates, according to the method described in Cochran.^17^ Average annual percentage change (AAPC) was calculated using Poisson regression. Models were calculated for all ages, and the pre-screening, screening and post-screening age ranges, using total CRC deaths as the response variable. Models included year as an explanatory variable and were adjusted for age-group and sex (for the models including both women and men), and the log of the population was included as an offset variable. The AAPC and associated 95% CI were calculated by taking the exponent of the year coefficient and the 95% of this coefficient from the model. Defining clear pre- and post-screening time periods was made difficult given the gradual piloting and introduction within Scotland. For clarity, AAPC was calculated for the period prior to any formal CRC screening taking place in Scotland (1990–99), and the period from which screening was first used through to the latest data available (2000–20). Models were calculated for all ages, and the pre-screening, screening and post-screening age ranges.

## Results

**Figure 1:**
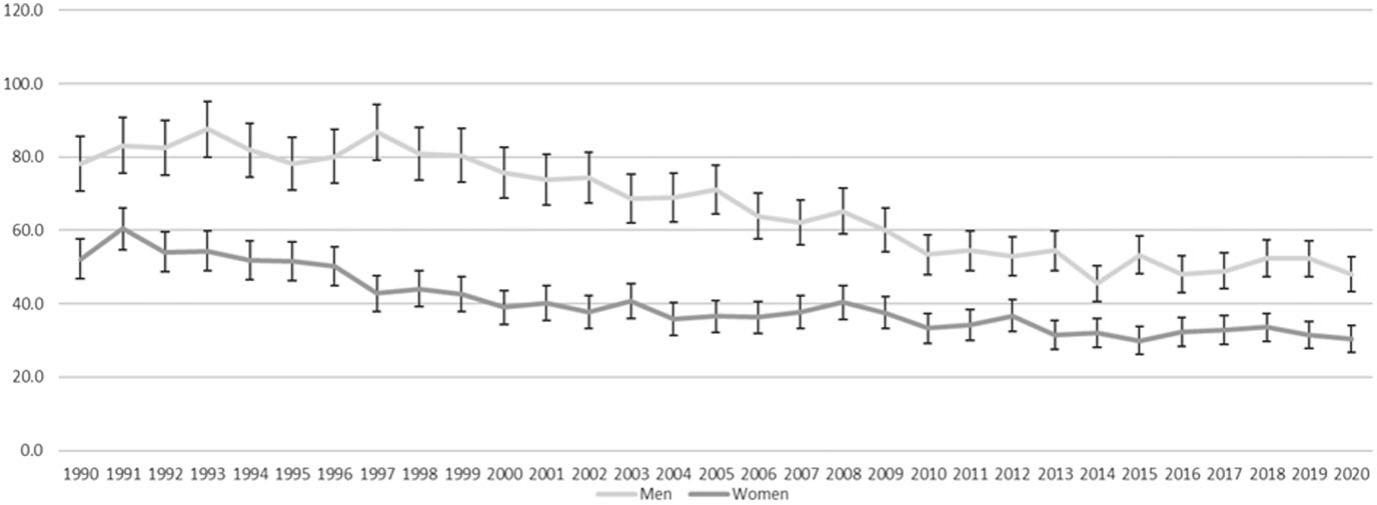
Age-standardized mortality per 100 000 for women and men of all ages from 1990 to 2020, with 95% CI shown as bars

**Figure 2:**
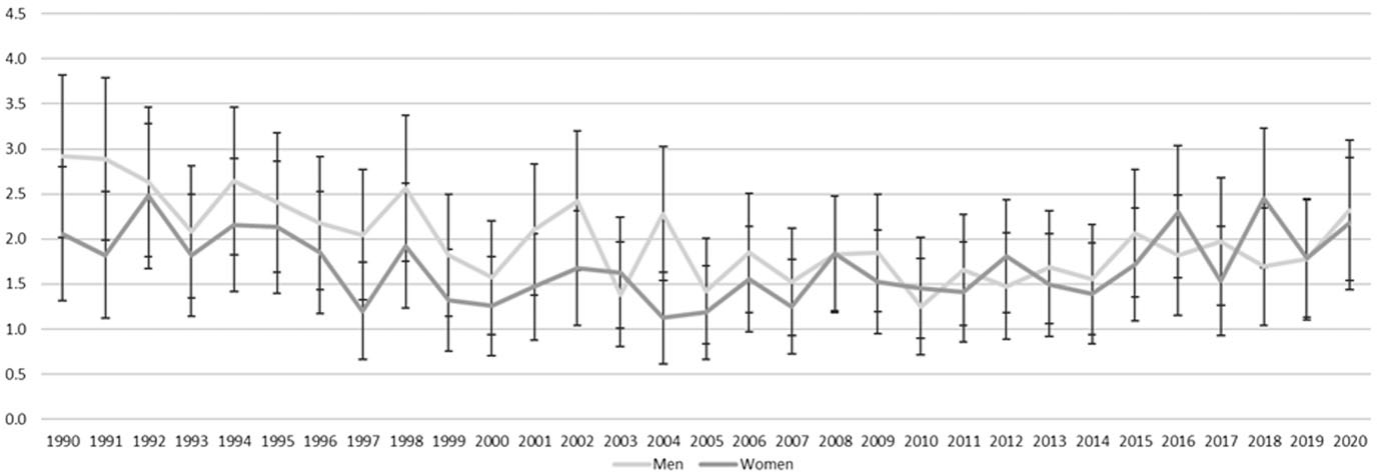
Age-standardized mortality per 100 000 for women and men <50 years from 1990 to 2020, with 95% CI shown as bars

**Figure 3:**
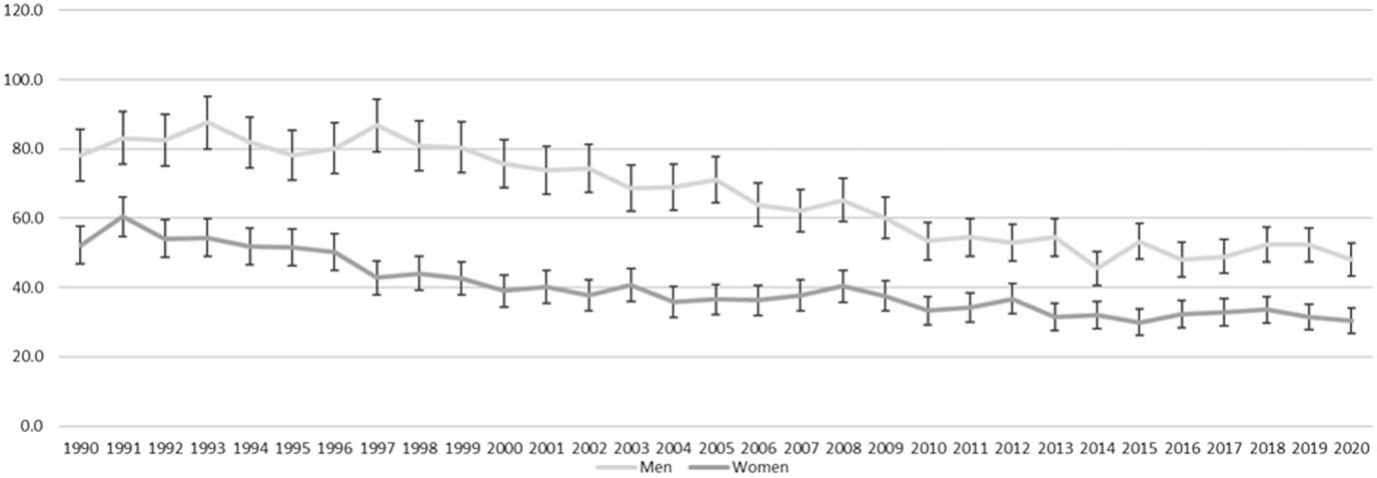
Age-standardized mortality per 100 000 for women and men aged 50–74 years from 1990 to 2020, with 95% CI shown as bars

**Figure 4:**
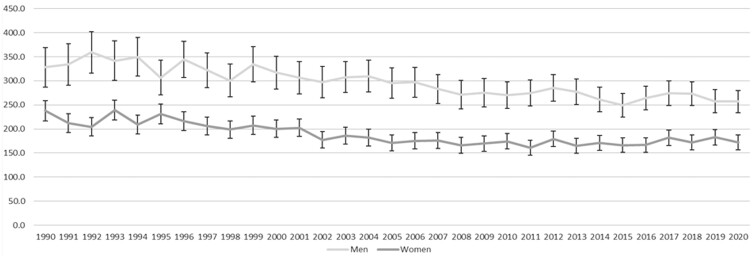
Age-standardized mortality per 100 000 for women and men >74 years from 1990 to 2020, with 95% CI shown as bars

Figure 1 shows that, in the whole population, CRC mortality in Scottish women and men declined from 1990 to 2020, but that the pattern of decline was not linear and differed between women and men. In women, the period between 1990 and 1999 was associated with a steady decline in CRC mortality from 38.3 per 100 000 to 32.3 per 100 000, but this was less pronounced after 2000. In men, there was little change from 1990 to 1999, when mortality was 54.8 and 55.4 per 100 000, respectively. Mortality then declined from 2000 when piloting of the SBoSP began and continued to decline through the subsequent roll-out to the whole population, which began in 2007. CRC mortality in men levelled out around 2014, at 38–41 deaths per 100 000. The same pattern was exaggerated in the screening age range (50–74 years) (figure 2) but was not seen in the pre-screening age range (figure 3) and was less pronounced in the post-screening age range (figure 4). Supplementary table S1 shows the AAPC for the time periods 1990–99 and 2000–20 in women and men in all age ranges together, and the pre-screening (<50 years), screening (50–74 years) and post-screening (>74 years) age ranges. For the time period 1990–99, the overall change was a reduction in mortality in women (AAPC: −2.1%, 95% CI: −2.8% to −1.4%), but not in men (−0.4%, 95% CI: −1.1% to 0.4%). The findings were similar in both the screening and post-screening age ranges, but there was no change in either sex in the pre-screening age range. For the time period 2000–20, the overall reduction in mortality was much less pronounced in women (−0.7%, 95% CI: −0.9% to −0.4%), than in men (−1.7%, 95% CI: −1.9% to −1.5%) and, in the screening age range, the reduction in mortality in women (−1.3%, 95% CI −1.7% to −0.9%) was around half that seen in men (−2.4%, 95% CI: −2.7% to −2.0%). In the post-screening group, reductions in mortality were also seen but were smaller (−0.5% in women and −1.1% in men). In contrast, an increase in mortality was seen in the pre-screening age range, and this was greater in women (+2.2%, 95% CI: +0.8% to +3.6%) than in men (+0.1%, 95% CI: −1.3% to +1.4%).

## Discussion

Our data show that, in Scotland, although population CRC mortality fell between 1990 and 2020, the patterns of decline differed markedly between women and men. Prior to initial gFOBT-based screening in 2000, there was a steady decline in mortality in women, but not in men. However, from the onset of screening, there was a pronounced fall in men until 2014, but a less pronounced fall in women in the screening age range but also in the post-screening age range. In the pre-screening age range, an increase in CRC mortality was seen in recent years, though only in women. These, and the many other discrepancies between women and men,^18^ are concerning and deserve further consideration.

The lower incidence of CRC explains the overall lower mortality in women but does not explain why the reduction in mortality in women differs over time from that in men. Temporal trends in CRC mortality can be attributed to changing incidence, stage at diagnosis, treatment, comorbidity burden and/or lifestyle factors That women experienced a fall in mortality between 1990 and 1999, which was not seen in men, suggests that improvements in detection and treatment are unlikely to have been responsible. However, women in Scotland have a healthier lifestyle than men in terms of diet, body mass index, general health and wellbeing and smoking since at least 1995^19^; this might explain the lower incidence of CRC^4^ and could be responsible for the woman-specific drop in mortality between 1990 and 2000. The relatively recent rise in mortality in those <50 years is likely to be related to the well-established increase in CRC incidence in young adults of both sexes,^20^ which may be caused by increasing body mass index and other lifestyle factors.^21^

The cessation of the mortality decline in 2014 in the screening age range is perhaps unsurprising since the effect of screening is likely to stabilize a few years after completion of roll-out. However, the most interesting question is why screening has been associated with such a small population effect in women compared with men. The changes in CRC mortality with time in both sexes indicates that, although mortality was falling in women at the onset of the screening programme, the same effect of screening as in men was not seen. Perhaps the increase in mortality in those women <50 years would also be seen in those 50–74 years but is being offset by screening. Alternatively, or additionally, the answer to this conundrum may be found in sex differences in the performance of CRC screening based on detection of f-Hb.

In women, CRC tends to be more right-sided than in men and thus less amenable to detection using FIT.^5^ Moreover, women are more likely to present with interval CRC (i.e. CRC diagnosed in the interval between screening episodes after a ‘negative’ screening test result),^5^^,^^6^ indicating that the f-Hb concentration is less diagnostically sensitive in women than in men.

The most likely explanation is related to the difference in f-Hb concentrations between women and men is because f-Hb concentrations in women are lower than in men.^22^ Thus, for any screening f-Hb concentration threshold, women have a lower positivity than men. In addition, irrespective of tumour stage and anatomical location, women with FIT screen-detected CRC have lower f-Hb concentrations than men.^9^

Why there should be such a marked sex difference in f-Hb concentration is unclear, but there are various cogent possibilities.^18^ Blood haemoglobin concentrations are lower in women; however, the difference becomes negligible after the menopause and the screening age range means that the majority of women participants are post-menopausal. Women have lower bowel transit times than men so that more degradation of f-Hb could occur prior to defaecation. Moreover, slowing of gut transit might be caused by constipation, which is more prevalent in women. Interestingly, women have greater colon lengths than men,^23^ another plausible reason for enhanced f-Hb degradation during colonic transit.

High f-Hb concentration is related to increased morbidity and mortality from causes other than CRC,^24^ implying that f-Hb concentration could be an indicator of poor health. Thus, individuals who have f-Hb detected on screening, but who have no obvious colorectal disease, may be at high risk of having, or developing, chronic disease. Possibly the lower f-Hb concentration in women may indicate less systemic inflammation than in men.

Nevertheless, quantitative FIT offer a possible rationale for the sex discrepancy in the mortality reduction in screening for CRC. Using a lower f-Hb concentration threshold for women than for men to trigger colonoscopy could go some way to correcting sex inequality, since it is likely that this would narrow the gap in terms of CRC detection and IC rates. However, increased numbers of colonoscopies would be needed for women and, since the positive predictive value (PPV) of FIT falls with decreasing f-Hb concentration thresholds,^25^ there could be an increase in the proportion of ‘false positive’ results in women.

Interestingly, a recent Finnish study found that a f-Hb concentration threshold of ≥25 μg Hb/g faeces for women and ≥70 μg Hb/g faeces for men resulted in similar CRC detection rates (0.16% in women and 0.18% in men) and almost identical PPV for CRC (6.4% and 6.6%).^26^ However, the positivities in women and men were 2.6% and 2.4%, considerably less than those at similar thresholds in Scotland, possibly due to lifestyle factors, and also because f-Hb concentrations depend on the FITused,^27^ so that Finnish data may not be transferable. A Swedish study investigated CRC screening employing differential f-Hb concentration thresholds for women and men of ≥40 µg Hb/g faeces and ≥80 µg Hb/g faeces.^28^ The results were contrasted to a threshold of ≥80 µg Hb/g faeces in both sexes. The positivity was 2.7% in both, but the PPV for CRC here was less in women (5.8%) than men (8.3%). ‘False positive’ FIT results were seen more often in women (24%) than men (17%); in 120 women diagnosed with CRC, 28 (23.3%) were found to have f-Hb ≤80 µg Hb/g faeces. It was concluded, however, that the additional CRC detected in women using the lower f-Hb concentration threshold compensated for the small increase in the cost of screening caused by different f-Hb thresholds for the sexes. In addition, the Swedish group have recently shown that using different f-Hb thresholds, the clinical sensitivity was higher, and the interval cancer rate was lower in women than in men.^29^

Both the incidence of CRC and the distribution of f-Hb concentrations vary from country to country,^30^ and even regionally in Scotland,^22^ and different f-Hb concentration thresholds are used to allow for colonoscopy capacities. Thus, the value of stratifying f-Hb thresholds by sex can only be studied by prospective studies. The evidence to date strongly suggests that a lower f-Hb concentration threshold in women would lead to increased detection of CRC. However, outstanding questions exist such as the extent of convergence of CRC detection rates, changes in PPV and the resulting harms of increasing the false positive rate, the impact on interval CRC and cost-effectiveness.

Since quantitative FIT are now used in the majority of programmes world-wide, few technical problems exist to create different f-Hb concentration thresholds for women and men. In the SBoSP, a threshold of ≥50 µg Hb/g faeces would give a positivity rate of 3.7% for women, the same as that in men at the threshold of ≥80 µg Hb/g faeces currently used for both sexes. To explore if equalizing positivity would equalize effectiveness in women and men, a consecutive series of women being offered a FIT could have their result reported as requiring further investigation at a threshold of ≥50 µg Hb/g faeces. Clearly, research carried out should not disadvantage any participant. In the case of the SBoSP, maintaining the threshold at ≥80 µg Hb/g faeces for men would achieve this laudable aim.

A way forward might be for all countries employing FIT-based CRC screening programmes to pilot different f-Hb concentration thresholds in women and men to equalize the positivity in both sexes. This would create an evidence base to inform the introduction of different thresholds for women and men, and CRC detection rates could be followed up by population-based CRC mortality statistics to study the effectiveness of this approach. Stratified approaches to CRC screening are now widely advocated,^31^ and this simple strategy could be in the vanguard.

## Supplementary Material

ckad029_Supplementary_Data

## Data Availability

Data are available upon reasonable request. Data may be available following consultation with Professor R.J.C. Steele: r.j.c.steele@dundee.ac.uk.
