# Supplementary material for: Sex variation in colorectal cancer mortality: trends and implications for screening
Source: Eur J Public Health. 2023 Feb 27;33(2):331–5. doi: 10.1093/eurpub/ckad029 (PMC10066495; doi:10.1093/eurpub/ckad029)
Supplement: ckad029_Supplementary_Data [file ckad029_supplementary_data.zip › ckad029_Supplementary_Data/ejph-2022-05-om-0283-File006.docx]

| **Cohort** | **1990 - 1999** | **2000 - 2020** |
| --- | --- | --- |
| All - all ages | -1.2% (-1.7% to -0.7%)* | -1.2% (-1.4% to -1.0%)* |
| Women - all ages | -2.1% (-2.8% to -1.4%)* | -0.7% (-0.9% to -0.4%)* |
| Men - all ages | -0.4% (-1.1% to 0.4%) | -1.7% (-1.9% to -1.5%)* |
| All - <50 years | -3.8% (-6.4% to -1.1%)* | 1.1% (0.1% to 2.1%)* |
| Women - <50 years | -4.0% (-7.8% to 0.0%)* | 2.2% (0.8% to 3.6%)* |
| Men - <50 years | -3.6% (-7.1% to -0.1%)* | 0.1% (-1.3% to 1.4%) |
| All - 50-74 years | -1.4% (-2.2% to -0.7%)* | -1.9% (-2.2% to -1.7%)* |
| Women - 50-74 years | -3.2% (-4.3% to -2.0%)* | -1.3% (-1.7% to -0.9%)* |
| Men - 50-74 years | -0.1% (-1.1% to 0.9%) | -2.4% (-2.7% to -2.0%)* |
| All - >74 years | -0.9% (-1.6% to -0.1%)* | -0.8% (-1.0% to -0.5%)* |
| Women - >74 years | -1.2% (-2.1% to -0.2%)* | -0.5% (-0.8% to -0.1%)* |
| Men - >74 years | -0.4% (-1.6% to 0.8%) | -1.1% (-1.5% to -0.8%)* |

**Supplementary Table 1.** Average annual percentage changes (AAPC), 95% CI and p-values < 0.05 for mortality over for the time periods 1990-1999 and 2000-2020 in all and in women and men of all ages, and in the pre-screening (<50 years), screening (50-74 years), and post-screening (>74 years) age ranges: * p-value < 0.05
